# Diagnostic markers for the detection of ovarian cancer in *BRCA1* mutation carriers

**DOI:** 10.1371/journal.pone.0189641

**Published:** 2017-12-15

**Authors:** Daphne Gschwantler-Kaulich, Sigrid Weingartshofer, Christine Rappaport-Fürhauser, Robert Zeilinger, Dietmar Pils, Daniela Muhr, Elena I. Braicu, Marie-Therese Kastner, Yen Y. Tan, Lorenz Semmler, Jalid Sehouli, Christian F. Singer

**Affiliations:** 1 Department of Obstetrics and Gynecology, Cancer Comprehensive Center, Medical University Vienna, Vienna, Austria; 2 Section for Clinical Biometrics, Center for Medical Statistics, Informatics, and Intelligent Systems (CeMSIIS), Medical University of Vienna, Vienna, Austria; 3 Department of Surgery, Medical University of Vienna, Vienna, Austria; 4 Department of Gynecology with Center for Oncological Surgery, European Competence Center for Ovarian Cancer, Charité - Campus Virchow-Klinikum, University Medicine of Berlin, Berlin, Germany; 5 QIMR Berghofer Medical Research Institute, Herston QLD, Australia; Ohio State University Wexner Medical Center, UNITED STATES

## Abstract

**Background:**

Screening for ovarian cancer (OC) in women at high risk consists of a combination of carbohydrate antigen 125 (CA125) and transvaginal ultrasound, despite their low sensitivity and specificity. This could be improved by the combination of several biomarkers, which has been shown in average risk patients but has not been investigated until now in female *BRCA* mutation carriers.

**Methods:**

Using a multiplex, bead-based, immunoassay system, we analyzed the concentrations of leptin, prolactin, osteopontin, insulin-like growth factor II, macrophage inhibitory factor, CA125 and human epididymis antigen 4 in 26 healthy wild type women, 26 healthy *BRCA1* mutation carriers, 28 wildtype OC patients and 26 OC patients with *BRCA1* mutation.

**Results:**

Using the ROC analysis, we found a high overall sensitivity of 94.3% in differentiating healthy controls from OC patients with comparable results in the wildtype subgroup (sensitivity 92.8%, AUC = 0.988; p = 5.2e-14) as well as in *BRCA1* mutation carriers (sensitivity 95.2%, AUC = 0.978; p = 1.7e-15) at an overall specificity of 92.3%.

The used algorithm also allowed to identify healthy *BRCA1* mutation carriers when compared to healthy wildtype women (sensitivity 88.4%, specificity 80.7%, AUC = 0.895; p = 6e-08), while this was less pronounced in patients with OC (sensitivity 66.7%, specificity 67.8%, AUC = 0.724; p = 0.00065).

**Conclusion:**

We have developed an algorithm, which can differentiate between healthy women and OC patients and have for the first time shown, that such an algorithm can also be used in *BRCA* mutation carriers. To clarify a suggested benefit to the existing early detection program, large prospective trials with mainly early stage OC cases are warranted.

## Introduction

Ovarian cancer is the most lethal cancer among gynaecological malignancies with a 5-year survival rate of patients diagnosed with advanced disease ranging from 20% to 25%. Only 20% of patients are diagnosed at stage I and II because of missing screening strategies [1].

While the lifetime risk of developing OC in the general population is about 1% to 2%, women with deleterious *BRCA* mutations have a cumulative lifetime risk of developing OC of approximately 45% in *BRCA1* carriers and 20% in *BRCA2* carriers [2,3]. While the early detection of breast cancer with the combination of mammography and MRI has a high success rate in this high risk population [4], the combination of transvaginal ultrasound (TVU) and CA125 for the early detection of ovarian cancer has suboptimal results. CA125 has a sensitivity of less than 60% in early stage OC with moderate improvement by the addition of TVU [5–9].

Furthermore, the incidence of serous tubal intraepithelial carcinomas (STIC) has been reported in a range from 0.6–7% in *BRCA* mutation carriers [10,11]. Therefore *BRCA1* or *BRCA2* mutation carriers are recommended to undergo risk reducing salpingo-oophorectomy (RRSO) by age 40 or after the completion of childbearing [12]. RRSO reduces the risk of OC by 85–90% and the risk of breast cancer by about 50% [13] and may also impact cancer-specific and overall mortality [14].

Studies have reported rates of RRSO in *BRCA* mutation carriers ranging from 12% to 78% underlining the importance of exact information about benefits and possible side-effects of this intervention in the presence of psychooncologists, because recommendations for OC screening for those who choose to forego or delay RRSO, are conflicting [15].

Therefore a better screening program for the early detection of OC is warranted for a better surveillance during the childbearing period, as a stop gap solution until mutation carriers decide to undergo RRSO and for those carriers deciding to forego RRSO.

Limited data exist regarding the combination of biomarkers to improve the early detection of OC in *BRCA* mutation carriers, although promising results have been reported using the combination of HE4 with CA125 [16,17]. Using a six biomarker panel consisting of macrophage inhibitory factor (MIF), prolactin, CA125, leptin, osteopontin and insulin like growth factor 2 (IGF2), Visintin et al reported an improved differentiation between disease free and ovarian cancer patients compared to CA125 alone in patients without a family history of OC (sensitivity of 95.3% vs 75%; specificity of 99.4% vs 95%) [18]. In this setting the combination of these six biomarkers with blood based gene expression achieved further improvement showing a sensitivity of 97.8% and a specificity of 99.6% [19].

To our knowledge, there exist no data evaluating the feasibility of the Milliplex 6-plex Ovarian Cancer Panel Kit combined with HE4 for the detection of ovarian cancer in *BRCA* mutation carriers. We therefore evaluated the serum- concentrations of these seven biomarkers in healthy non carriers, healthy *BRCA1* mutation carriers, patients with sporadic OC and *BRCA1* mutation carriers with OC.

## Materials and methods

### Ethics, consents

The ethical board of the Medical University of Vienna approved this study. Patients had to sign an informed consent prior to inclusion into the study.

### Patient population

#### Ovarian cancer group

The disease group (n = 54) included wildtype women with newly diagnosed OC (n = 28) and *BRCA1* mutation carriers with OC (n = 26). Eleven samples of wildtype OC patients were obtained from the biobank of the Medical University of Vienna and 17 samples of wildtype OC patients as well as 26 samples of *BRCA1* mutation carriers with OC from the tumorbank ovarian cancer (TOC) of the Charité, Medical University of Berlin. Median age in the wildtype group was 57.6 yrs (33.6–82.1) and in the *BRCA1* mutation carrier group 53.0 yrs (41.0–78.0). All samples were collected prior to surgery. Table 1 shows detailed information about histology and stage of the disease.

**Table 1:** Patients characteristics of the healthy control group (wildtype and *BRCA1*) and patients tumor characteristics of the group of ovarian cancer patients (wildtype and *BRCA1*).

	Wildtype healthy (n = 26)	*BRCA1* healthy (n = 26)	Wildtype OC (n = 28)	*BRCA1* OC (n = 26)
**Median age** (yrs) (range)	67.1 (60.7–72.4)	36.4 (26.4–61.7)	57.6 (33.6–82.1)	53.0 (41.0–78.0)
**OC histology**				
serous			23 (82.1%)	22 (84.6%)
mucinous			0 (0.0%)	1 (3.8%)
endometroid			2 (7.2%)	1 (3.8%)
NA			3 (10.7%)	2 (7.8%)
**Grading**				
G1			0 (0.0%)	0 (0.0%)
G2			6 (21.4%)	7 (26.9%)
G3			20 (71.4%)	19 (73.1%)
NA			2 (7.2%)	0 (0.0%)
**FIGO**				
1a			0 (0.0%)	1 (3.8%)
1b			0 (0.0%)	0 (0.0%)
1c			0 (0.0%)	2 (7.8%)
2a			1 (3.6%)	0 (0.0%)
2b			1 (3.6%)	0 (0.0%)
2c			1 (3.6%)	0 (0.0%)
3a			1 (3.6%)	0 (0.0%)
3b			6 (21.4%)	0 (0.0%)
3c			14 (50.0%)	19 (73.0%)
4			4 (14.2%)	4 (15.4%)

Abbreviations: OC = ovarian cancer

#### Control group

The healthy control group (n = 52) included age-matched sera from healthy wildtype women (n = 26) and healthy *BRCA1* mutation carriers (n = 26) obtained from the biobank of the Medical University of Vienna. Median age in the healthy wildtype group was 67.1 yrs (60.7–72.4) and 36.4 yrs (26.4–61.7) in healthy *BRCA1* mutation carriers. We included only *BRCA1* mutation carriers who have not undergone risk reducing surgery. (Table 1)

### Sample collection

Ten mL of peripheral blood was drawn from subjects using standardized phlebotomy procedures [20]. Samples were processed within two to four hours using guidelines set by the National Cancer Institute Inter-Group Specimen Banking Committee and stored at -80°C in the sera bank.

### Multiplex analysis

Serum determinations for MIF, leptin, prolactin, OPN, CA125, and IGF II were performed using the beadlyte 6-plex Ovarian Cancer Panel Kit and a kit for the analysis of HE4, both from Millipore, according to the manufacturers instructions [18,19]. The 6-plex Ovarian Cancer Panel Kit included two panels: one for prolactin, leptin, OPN, MIF and CA125 (Beadlyte 5-plex Ovarian Cancer Panel) and a separate panel for IGF-II (Beadlyte Anti-Human IGF-II Bead Set). Because of data suggesting that HE4 plays a role in OC, we added a separate kit for the detection of HE4 (Beadlyte Anti-Human HE4 Bead Set).

### Statistical analysis

Missing Luminex values, i.e. values below analyte-specific detection limits, were imputed with analyte-corresponding PBS-values divided by square root of two. The geometric mean of two calibrator samples were used as reference to level plate specific differences and all values were log-2 transformed to get (near) parametric distributions.

Statistical differences over all four/five groups (CTRL WT, CTRL BRCA, Ca WT, and Ca BRCA) were calculated by one-way analysis of variance (ANOVA) with subsequent–if significant–post-hoc tests according Tukey's ‘Honest Significant Difference’ method. Multivariable discriminative models were built by logistic regressions and a cut-off defined by maximizing specificity and sensitivity simultaneously (R-package OptimalCutpoints). Receiver Operating Characteristic (ROC) curves, corresponding area under these ROC curves (AUC), and p-values are presented. (Two-sided) p-values below 0.05 were considered as statistically significant. All analyses were performed with R version 3.3.3 [21] and R-packages: ROCR v1.0–7 [22] and OptimalCutpoints v1.1–3 [23].

## Results

### Differentiation between healthy women and OC patients

We performed multiplex analysis and evaluated the serum determination of the seven biomarkers (CA125, MIF, Leptin, OPN, Prolactin, IGF2 and HE4) in healthy wildtype women, healthy women with *BRCA1* germline mutation, wildtype OC patients and *BRCA1* mutation carriers with OC. Since 17 samples of patients with wildtype OC and 26 samples of *BRCA1* mutation carriers with OC came from one institution (tumorbank ovarian cancer (TOC) of the Charité, Medical University of Berlin), we performed a test for interaction in order to identify possible laboratory or sampling bias. We found no significant difference for CA125, leptin, OPN, MIF, IGF2 and HE4. However, the prolactin levels in the respective subgroups differed significantly between the two centers and we therefore excluded prolactin from our algorithm (see S1 Fig).

**Fig 1:**
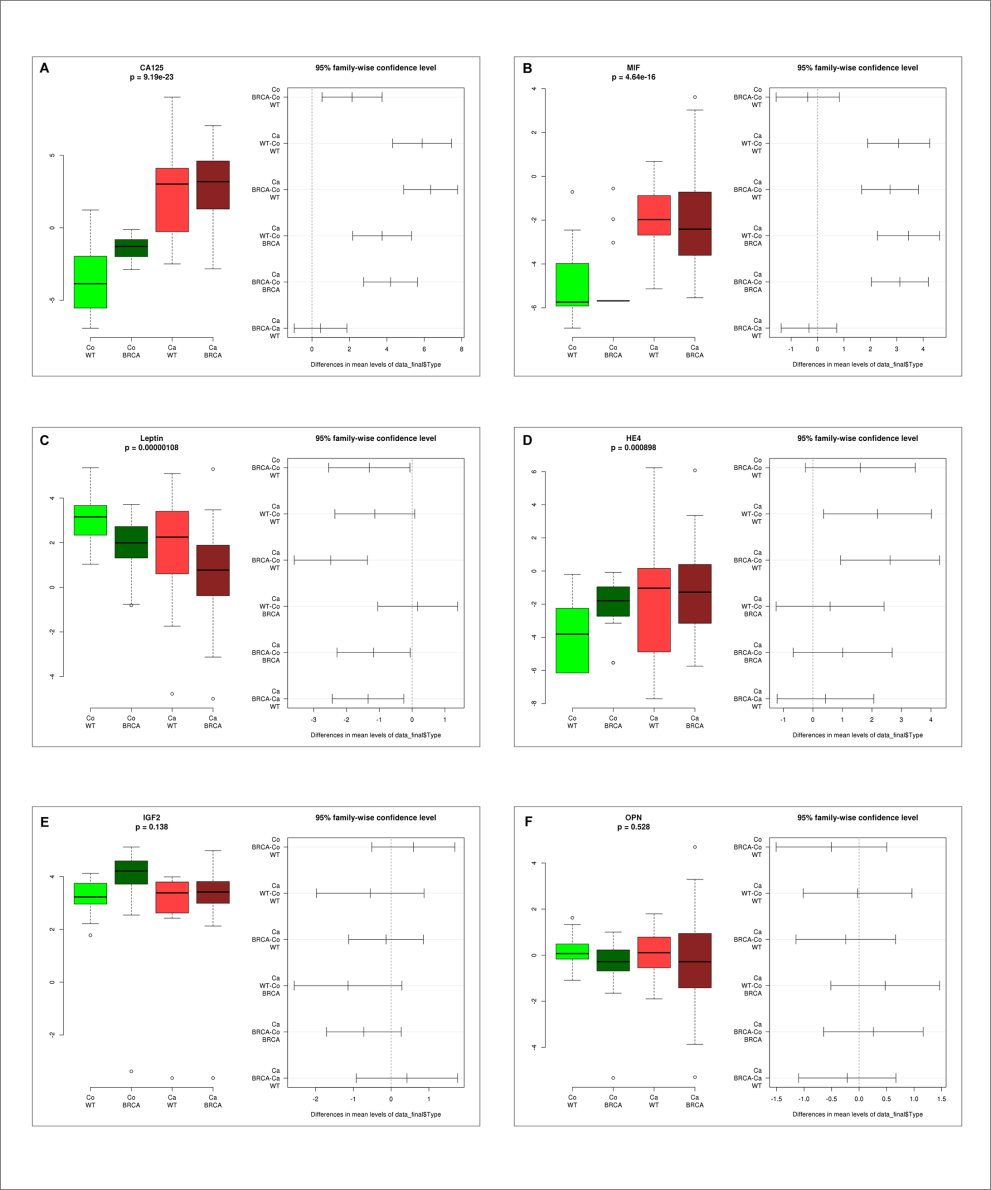
Serum levels of the six biomarkers. Different serum levels of CA125 (A), MIF (B), Leptin (C), HE4 (D), IGF2 (E), OPN (F) in the four groups (Co WT = healthy wildtype, Co BRCA = healthy *BRCA1* mutation carriers, CaWT = wildtype ovarian cancer patients, CaBRCA = *BRCA1* mutation carriers with ovarian cancer).

The individual serum levels of the evaluated biomarkers are shown in Fig 1A–1F.

CA125 (Fig 1A) and MIF (Fig 1B) were shown to be the best single markers to differentiate between healthy women and OC patients in wildtype as well as *BRCA1* mutation carriers (MIF: p = 4.64e-16; CA-125: p = 9.19e-23, respectively) followed by Leptin (p = 0.00000108; Fig 1C). While different levels of HE4 (Fig 1D) lead to a significant difference between healthy wildtype women and wildtype OC patients (p = 0.000898), we found no significant difference in the HE4 levels of healthy *BRCA1* mutation carriers and those with OC. We found no significant differences between the four groups comparing their levels of IGF2 (P = 0.138; Fig 1E) or OPN (p = 0.528; Fig 1F).

We then adjusted our results for age and looked at correlations between the level of the investigated biomarkers and age (S2–S5 Figs, Table 2). We found a positive correlation of MIF with age (R = 0.42, p<0.05) and a negative correlation of IGF2 with age (R = -0.35, p<0.1) in the wildtype control group. We found no such correlations in the *BRCA1* groups where the difference in age between healthy mutation carriers and those with OC were significant.

**Table 2:** Correlations of the levels of the investigated biomarkers with age in the four study groups.

	age	CA125	MIF	Leptin	HE4	IGF2	OPN	Prolactin
Co WT	67,1		**0,42**			***-0*,*35***		
Co *BRCA1*	36,4							
CA WT	57,6				0,49	-0,50	0,47	
CA *BRCA1*	53,0							

**bold:** p<0.05, ***bold*, *italic***: p<0.100, black: not sign.

### Differentiation between wildtype women and *BRCA1* mutation carriers

We then investigated if there are differences in the levels of these six biomarkers between wildtype women and *BRCA1* mutation carriers. In the healthy cohort, we found significant higher levels of CA125 (Fig 1A) and lower levels of Leptin (Fig 1E) in *BRCA1* mutation carriers. In the OC cohort, Leptin was the only biomarker with significantly different (lower) levels between wildtype OC patients and *BRCA1* mutation carriers (Fig 1E).

### ROC Analysis

**Fig 2:**
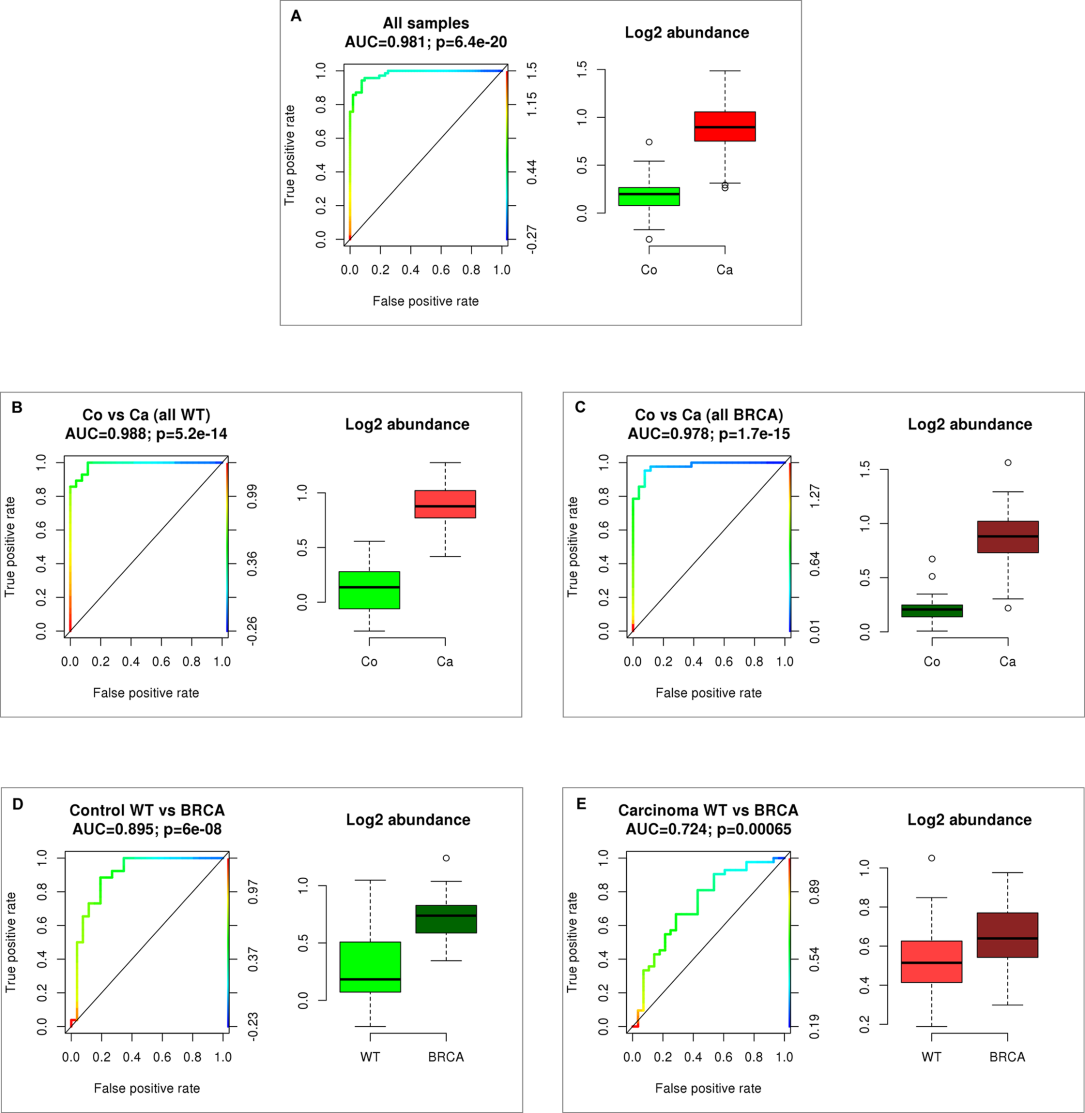
Sensitivity and specificity. Sensitivity and specificity of the combination of five biomarkers (CA125, MIF, Leptin, HE4, OPN) in differentiating between healthy women and OC patients (A), healthy wildtype women and wildtype OC patients (B), healthy *BRCA1* mutation carriers and *BRCA1* mutation carriers with OC (C), healthy wildtype women and healthy *BRCA1* mutation carriers (D) and wildtype OC patients and *BRCA1* mutation carriers with OC (E).

Since IGF2 levels were not available from all patients, we restricted our algorithm to five biomarkers (MIF, Leptin, CA125, OPN and HE4) using area under the receiver operating charactersistic (ROC) curves (AUCs). When we evaluated the value of the five biomarkers in differentiating between healthy women and OC patients regardless of their mutation status, we found a sensitivity of 94.3% (AUC = 0.981; p = 6.4e-20; Fig 2A). We then calculated the ROC AUC separetely for the wildtype subgroup showing a sensitivity of 92.8% (AUC = 0.988; p = 5.2e-14; Fig 2B) and the subgroup of *BRCA1* mutation carriers resulting in a sensitivity of 95.2% (AUC = 0.978; p = 1.7e-15; Fig 2C) at an overall sensitivity of 92.3%.

We then investigated if these 5 biomarkers are able to differentiate between wildtype and *BRCA1* mutation carriers and found a sensitivity of 88.4% at a specificity of 80.7% (AUC = 0.895; p = 6e-08) in the healthy subgroup (Fig 2D) and a sensitivity of 66.7% at a specificity of 67.8% (AUC = 0.724; p = 0.00065) in the subgroup of OC patients (Fig 2E).

## Discussion

To date, screening for OC in women at high risk consists of the combination of CA125 and TVU, although evidence is insufficient to demonstrate that these tests provide a survival benefit [8,9]. Improvement of OC screening strategies in high risk women is urgently warranted not only for *BRCA1* mutation carriers who decide to postpone or forego RRSO, but also for women with a family history who should follow early detection programs for breast and ovarian cancer.

We here present the first study comparing the serum levels of a combination of five biomarkers (MIF, leptin, OPN, CA125 and HE4) in healthy wildtype women, healthy *BRCA1* mutation carriers, wildtype OC patients and *BRCA1* mutation carriers with OC.

Our data demonstrate that an algorithm based on these five proteins is able to significantly differentiate between healthy and OC patients in wildtype patients as well as *BRCA1* mutation carriers.

In average risk women, a wide range of diagnostic approaches like panels of biomarkers, algorithms, ultrasound and other imaging methods have been investigated to improve the early detection of OC [24–28].

Concentrating on biomarkers, HE4 has been reported to be superior to CA125 in separating benign, borderline ovarian tumors, cancers of the fallopian tubes, as well as early stage epithelial OC [29–39]. Together with CA125 and menopausal status it has been incorporated into the Risk of Ovarian Malignancy Algorithm (ROMA) [40] and in combination with CA125-II, apolipoprotein A-1, follicle stimulating hormone, and transferrin into the Overa-Test in order to discern malignant from benign pelvic masses [41].

Osteopontin is another interesting biomarker, which has been shown to ameliorate the discriminating ability between benign and malign pelvic masses when combined with HE4 and CA125 [42]. Furthermore, El-Tanani MK et al demonstrated that *BRCA1* mutation lead to OPN overexpression resulting in proliferation of breast cancer cells in a rat mammary model system [43], thus OPN could also be an important biomarker in the development of OC in *BRCA1* mutation carriers.

There is some evidence that lower levels of leptin [44] and higher levels of prolactin [45] might be associated with increased risk of ovarian cancer.

The combination of the above mentioned six biomarkers (MIF, OPN, CA125, IGF II, leptin and prolactin) has been shown to improve differentiation between disease free and ovarian cancer patients compared to CA125 alone in patients without a family history of OC [18,19]. Other studies reported similar benefits when combining these six biomarkers with p53 [46] or interleukin 18 (IL-18) and fibroblast growth factor 2 (FGF-2) [47].

Although these developments seem to be promising, recent data suggest that CA125 is still the best single marker for the early detection of OC [48–50] in average risk women and limited data exists regarding the use of these biomarkers as OC screening in high risk women.

In high risk women, it has been suggested that higher cut-off levels and frequent CA125 testing could improve the low sensitivity and specificity of the current early detection program [51]. Our data, which show significantly higher levels of CA125 in healthy *BRCA1* mutation carriers and lower levels of leptin when compared to healthy wildtype women, confirm the suggested necessity of individual adjustment of CA125 cut-off levels.

Our study has several limitations like the small sample size and the possible laboratory or sampling bias, because samples have been obtained from two different biobanks. Although all OC samples have been collected prior to surgery following a standardized protocol in the two centers, we performed a test for interaction and found no significant difference for CA125, leptin, OPN, MIF, IGF2 and HE4. However, the prolactin levels differed significantly between the two centers and we therefore excluded prolactin from our algorithm.

Furthermore our study is limited by the difference in age between healthy *BRCA1* mutation carriers and those with OC as age could impact the expression of biomarkers. While we found a positive correlation of the level of MIF in the healthy wildtype group, we found no such correlation in healthy *BRCA1* mutation carriers or those with OC. We found slightly higher levels of MIF in the healthy wildtype group (oldest cohort) compared to the healthy *BRCA1* mutation carriers (youngest cohort), but in both OC groups (WT and *BRCA1*) the levels of MIF were significantly higher than in the healthy control groups. Furthermore, we found a negative correlation between the levels of IGF2 and age and therefore suggest that the higher levels of IGF2 (p = n.s.) in healthy *BRCA1* mutation carriers result from the younger age in this group.

Taken together we have developed an algorithm, which can differentiate between healthy women and OC patients with a sensitivity of 94.3% and a specifity of 92.3% and have for the first time shown, that such an algorithm can also be used in *BRCA* mutation carriers with a sensitivity of 95.2% and a specifity of 92.3%.

However, to verify if this algorithm could improve the early detection of OC in high risk women, larger prospective trials with mainly early stage OC cases are warranted.

## Supporting information

S1 Fig Serum levels of prolactin. Different serum levels of Prolactin with significant differences between samples from the two biobanks (Vienna and Berlin). We therefore excluded Prolactin from our calculations.
(PNG)

S2 Fig Correlations of the investigated biomarkers with age in the Co WT group. Correlation coefficient (R); °p<0.1 (not sign.); * p<0.05; ** p <0.01; *** p <0.001(Spearman).
(TIFF)

S3 Fig Correlations of the investigated biomarkers with age in the CA WT group. Correlation coefficient (R); °p<0.1 (not sign.); * p<0.05; ** p <0.01; *** p <0.001(Spearman).
(TIFF)

S4 Fig Correlations of the investigated biomarkers with age in the Co BRCA1 group. Correlation coefficient (R); °p<0.1 (not sign.); * p<0.05; ** p <0.01; *** p <0.001(Spearman).
(TIFF)

S5 Fig Correlations of the investigated biomarkers with age in the CA BRCA1 group. Correlation coefficient (R); °p<0.1 (not sign.); * p<0.05; ** p <0.01; *** p <0.001(Spearman).
(TIFF)
