# Correction: Diagnostic markers for the detection of ovarian cancer in *BRCA1* mutation carriers

**DOI:** 10.1371/journal.pone.0196142

**Published:** 2018-04-17

**Authors:** Daphne Gschwantler-Kaulich, Sigrid Weingartshofer, Christine Rappaport-Fürhauser, Robert Zeillinger, Dietmar Pils, Daniela Muhr, Elena I. Braicu, Marie-Therese Kastner, Yen Y. Tan, Lorenz Semmler, Jalid Sehouli, Christian F. Singer

The fourth author's name is spelled incorrectly. The correct name is: Robert Zeillinger. The correct citation is: Gschwantler-Kaulich D, Weingartshofer S, Rappaport-Fürhauser C, Zeillinger R, Pils D, Muhr D, et al. (2017) Diagnostic markers for the detection of ovarian cancer in *BRCA1* mutation carriers. PLoS ONE 12(12): e0189641. https://doi.org/10.1371/journal.pone.0189641
