# Supplementary figures and images for: Diagnostic markers for the detection of ovarian cancer in BRCA1 mutation carriers
Source: PLoS One. 2017 Dec 15;12(12):e0189641. doi: 10.1371/journal.pone.0189641 (PMC5731824; doi:10.1371/journal.pone.0189641)

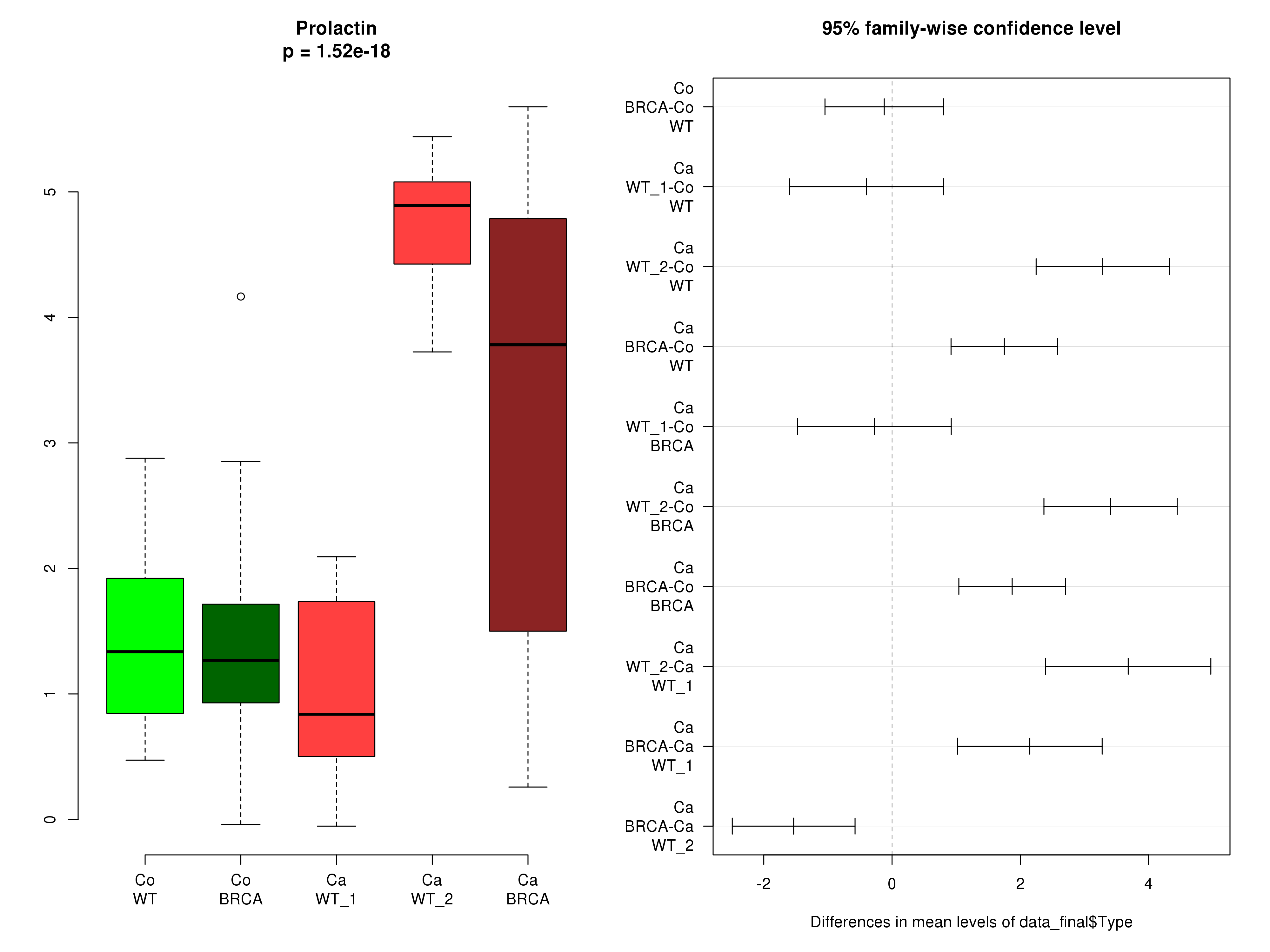

Supplement: S1 Fig — Different serum levels of Prolactin with significant differences between samples from the two biobanks (Vienna and Berlin). We therefore excluded Prolactin from our calculations. (PNG) [file pone.0189641.s001.png]

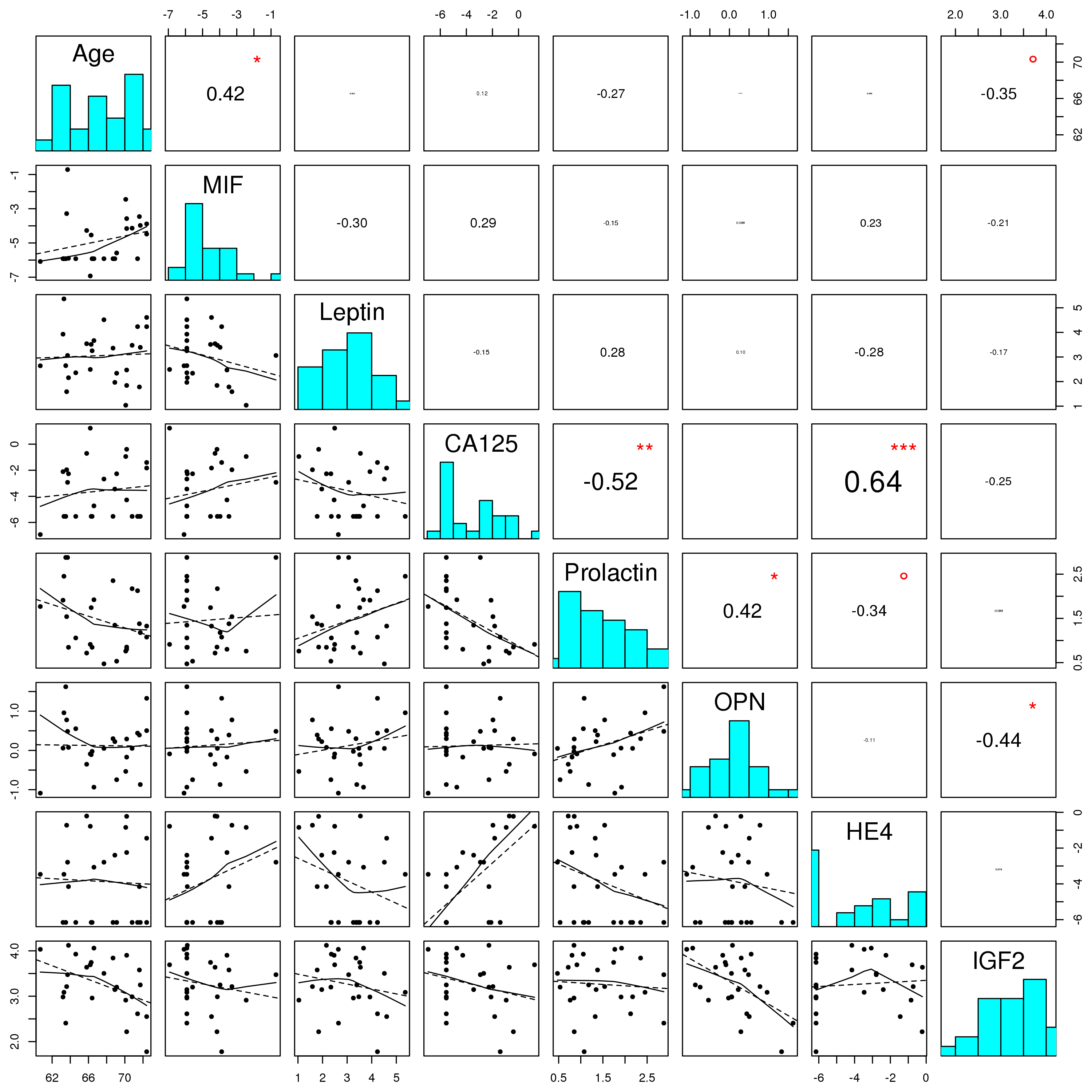

Supplement: S2 Fig — Correlation coefficient (R); °p<0.1 (not sign.); * p<0.05; ** p <0.01; *** p <0.001(Spearman). (TIFF) [file pone.0189641.s002.tiff]

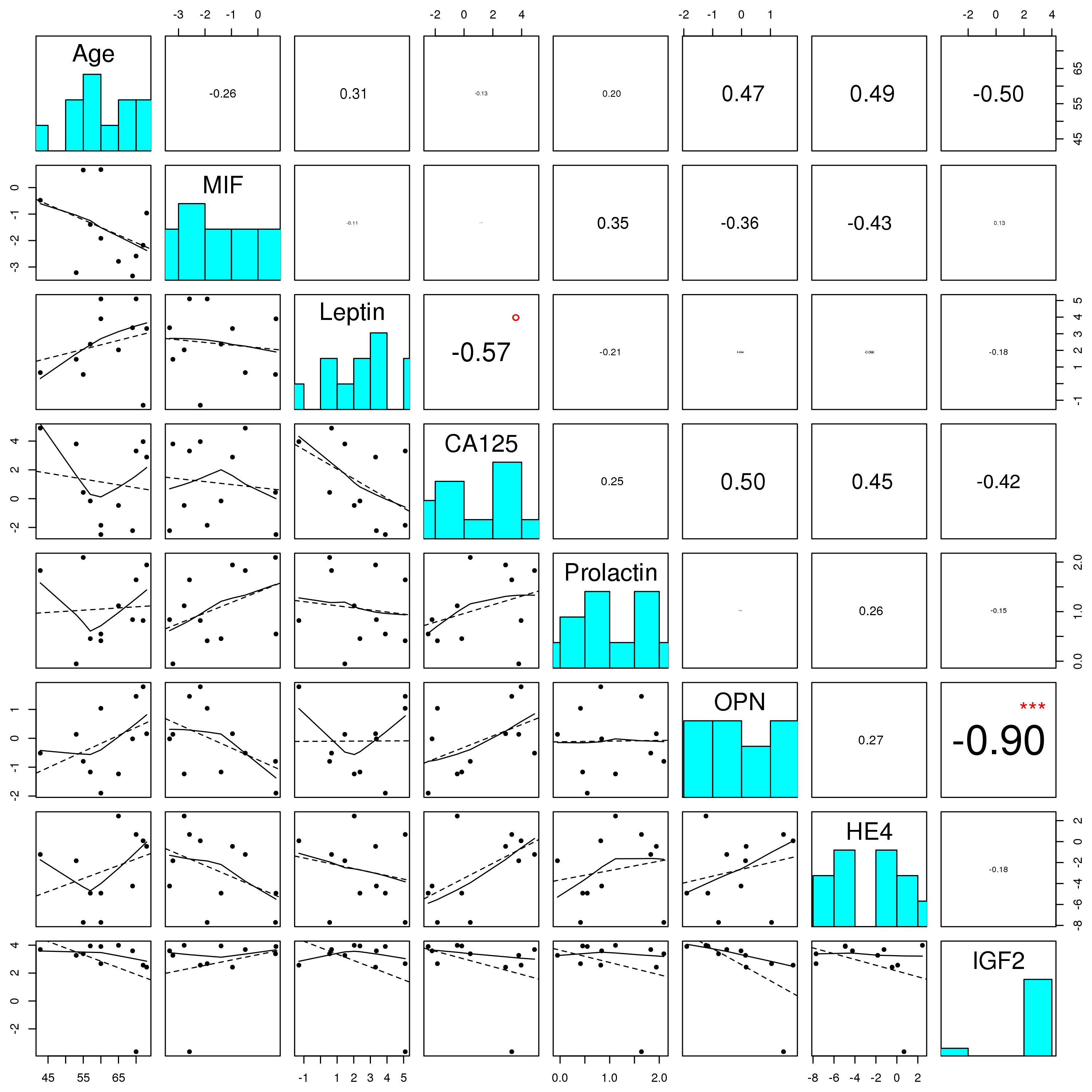

Supplement: S3 Fig — Correlation coefficient (R); °p<0.1 (not sign.); * p<0.05; ** p <0.01; *** p <0.001(Spearman). (TIFF) [file pone.0189641.s003.tiff]

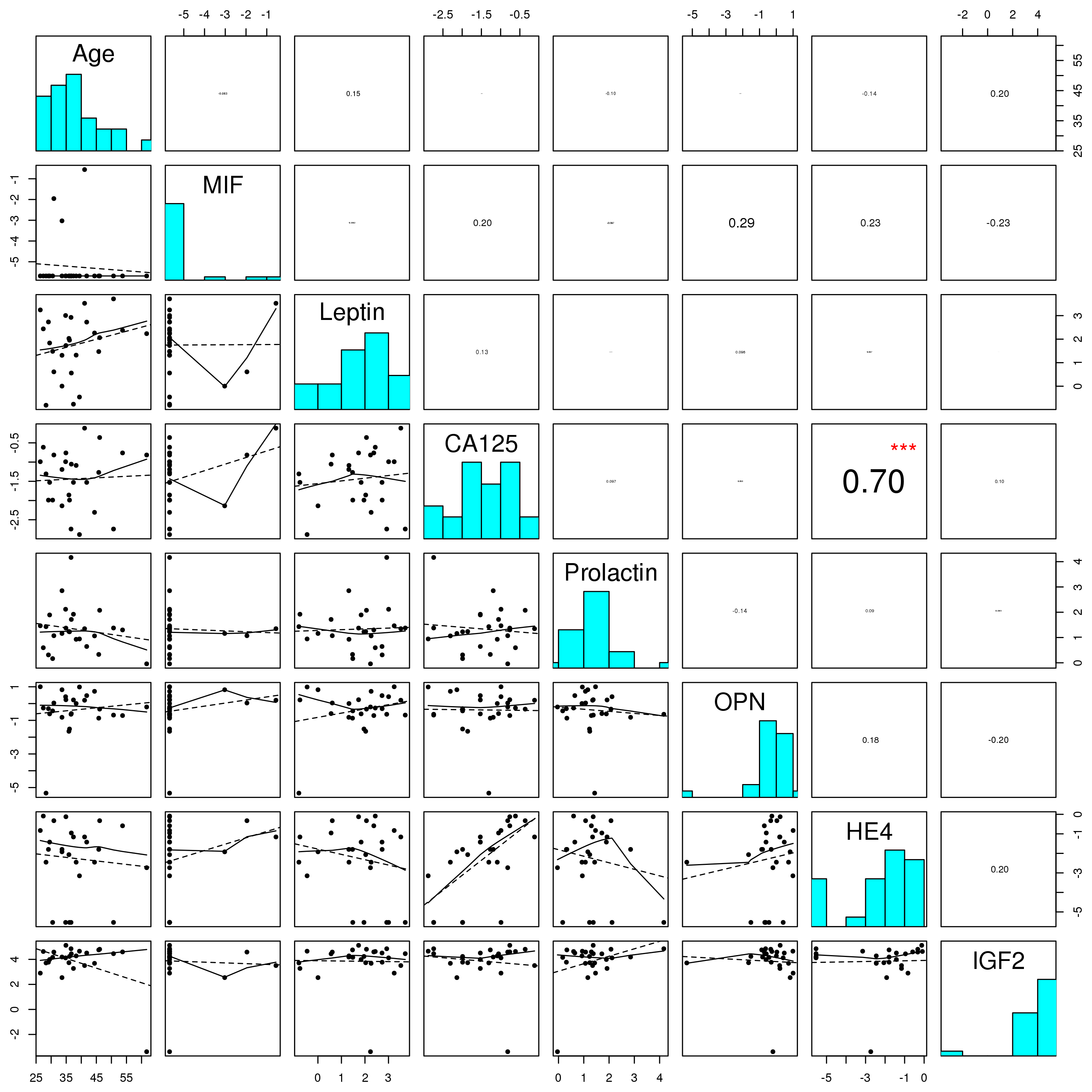

Supplement: S4 Fig — Correlation coefficient (R); °p<0.1 (not sign.); * p<0.05; ** p <0.01; *** p <0.001(Spearman). (TIFF) [file pone.0189641.s004.tiff]

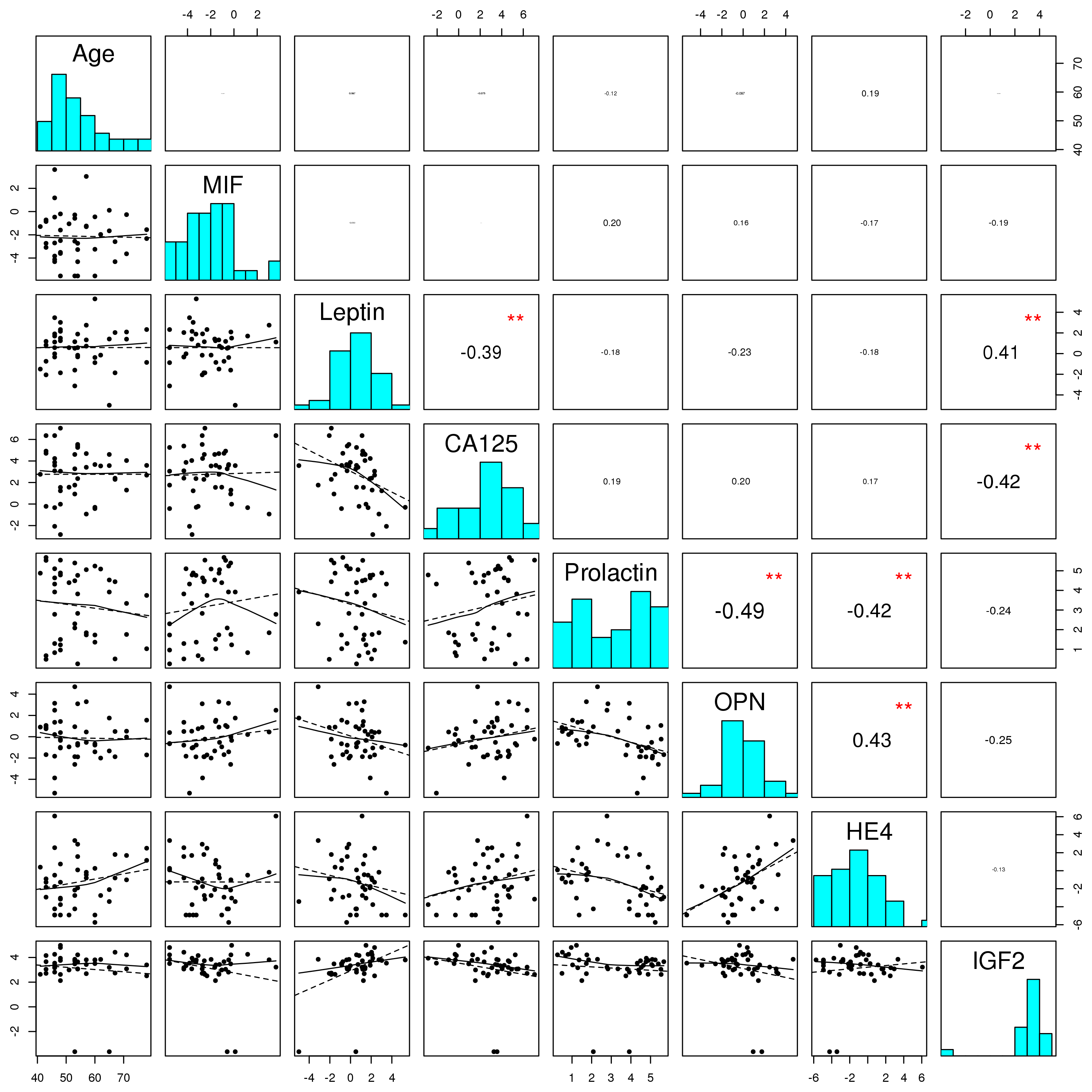

Supplement: S5 Fig — Correlation coefficient (R); °p<0.1 (not sign.); * p<0.05; ** p <0.01; *** p <0.001(Spearman). (TIFF) [file pone.0189641.s005.tiff]
